# Toxicity of emulsions, high and low energy nanoemulsions of orange essential oil and d-limonene to *Drosophila suzukii*, and selectivity to *Pachycrepoideus vindemmiae*

**DOI:** 10.1007/s13205-026-04833-9

**Published:** 2026-05-14

**Authors:** Carolina Bojacá López, Raiany Soares de Lima, Lucas Simões de Assis, Juliano Elvis de Oliveira, María Alice Martins, Rocío Yanet Farro-Barbarán, Khalid Haddi

**Affiliations:** 1 Laboratory of Molecular Entomology and EcoToxicology, Entomology Department, Federal University of Lavras, Lavras, MG Brazil; 2 Department of Chemical and Materials Engineering, Federal University of Lavras, Lavras, 37203-202 Brazil; 3 Laboratório Nacional de Nanotecnologia para o Agronegócio (LNNA), Embrapa Instrumentação, São Carlos, SP 13561-206 Brasil

**Keywords:** Spotted wing drosophila, Bioinsecticide, Nanoemulsion, Pest control

## Abstract

Sweet orange essential oil (*Citrus sinensis*) and its major component d-limonene were formulated as emulsions and low- and high-energy nanoemulsions and evaluated for toxicity against adults, larvae, and pupae of *Drosophila suzukii*, as well as for selectivity toward the pupal parasitoid *Pachycrepoideus vindemmiae*. High-energy nanoemulsions produced smaller and more homogeneous droplets (221–289 nm; PDI 0.210–0.255) compared with low-energy nanoemulsions (617–796 nm; PDI 0.548–0.644), indicating improved dispersion and potential stability. Toxicity assays revealed that the pure essential oil and d-limonene were the most toxic to adult *D. suzukii* (LC_50_ = 1.7 wt% and 0.54 wt%, respectively), followed by high-energy nanoemulsions, emulsions, and low-energy nanoemulsions. Although pure compounds showed slightly higher toxicity, high-energy nanoemulsions maintained comparable efficacy, suggesting improved delivery without major loss of activity. Larval mortality did not exceed 40% across treatments, whereas pupae were markedly more susceptible. High-energy nanoemulsions of both orange essential oil and d-limonene produced the highest pupal mortality (around 85%), followed by low-energy nanoemulsions (72–77.5%) and emulsions (50–75%). In addition, emulsified and nanoemulsified formulations induced developmental abnormalities in emerged adults, with malformation rates reaching 30%. Selectivity assays demonstrated low toxicity of all formulations to adult *P. vindemmiae*, with mortality generally below 20% and a negligible reduction of parasitism activity in most treatments. Overall, the formulation method strongly influenced physicochemical properties and insecticidal performance. High-energy nanoemulsions enhanced efficacy, particularly against pupae, while maintaining low toxicity to a key parasitoid. These findings indicate that nanoformulated citrus essential oils represent promising selective and environmentally compatible tools for integrated management of *D. suzukii*.

**Supplementary Information:**

The online version contains supplementary material available at 10.1007/s13205-026-04833-9.

## Introduction

The spotted wing drosophila (SWD), *Drosophila suzukii* (Matsumura 1931)(Diptera: Drosophilidae), is a polyphagous pest that affects the fruits of a wide range of host species. Native to Southeast Asia, it has expanded its geographic range since 2008 to Europe (Calabria et al. 2012), North and South America (Deprá et al. 2014), and Africa (Boughdad et al. 2021). Current management strategies for *D. suzukii* heavily depend on chemical controls (Garcia et al. 2022). However, this reliance on synthetic pesticides raises significant concerns due to their ecological and health implications, notably the toxicity to non-target organisms and the propensity for pests to develop resistance (Deans and Hutchison 2022; Gress and Zalom 2019). This backdrop has intensified the exploration of viable natural alternatives, such as biopesticides known for their potent insecticidal properties, low toxicity to non-target species, and safety to humans and the environment.

In this sense, biopesticides of plant origin, especially essential oils (EOs), have gained prominence in research as they are bioactive, biodegradable, and environmentally safe (Giunti et al. 2022), in addition to their raw material availability and good cost-benefit ratio. Extracted from various parts of plants, such as roots, stems, and leaves, essential oils are oily, lipophilic, volatile compounds that mainly include terpenes and terpenoids, aliphatic compounds, and aromatic substances (Mahawer et al. 2022). EOs have attracted attention for their insecticidal activity manifested in the form of acute toxicity, repellency, antifeeding, oviposition deterrence, and inhibition of development and reproduction (Isman 2020; Pavela and Benelli 2016).

Recent studies have explored the effectiveness of various essential oils and their components in managing *D. suzukii* (Dam et al. 2019; Dos Santos et al. 2024). Terpenes and phenylpropanoids have proven effective not only in inducing acute toxicity but also in causing oxidative stress and histopathological alterations in these pests, offering a potentially selective approach for their control (de Souza et al. 2024). Similarly, the essential oil of *Illicium verum* has shown significant toxicity and inhibition of acetylcholinesterase in *D. suzukii*, also causing notable histopathological changes in the insect’s tissues (de Souza et al. 2022a, b). Additionally, essential oils from geranium (*Pelargonium graveolens*), dill (*Anethum graveolens*), and Scots pine (*Pinus sylvestris*) have displayed considerable insecticidal and repellent effects against *D. suzukii*. Notably, geranium has demonstrated a deterrent effect, repelling egg-laying females for 4 days even at the lowest concentration applied (Bošković et al. 2023). Another study found that essential oils from Several *Baccharis* species, and their main component, d-limonene, exert significant insecticidal effects and deter oviposition in *D. suzukii*. These oils caused adult mortality exceeding 80% at a concentration of 80 mg L^−1^, with efficacy comparable to that of spinetoram (75 mg L^−1^). Moreover, d-limonene and the essential oils from *Baccharis* displayed lower LC_50_ and LC_90_ values than those of spinosyn and azadirachtin (de Souza et al. 2021).

Although essential oils are recognized for their substantial potential as active ingredients in biopesticides, they face challenges related to their physicochemical characteristics; high volatility, limited water solubility, and rapid degradation pose significant barriers to their effective application in practical settings (Huo et al. 2024). Furthermore, standardizing the chemical composition of essential oils presents difficulties, as samples labeled as identical can vary substantially in their chemical profiles due to factors such as cultivation conditions, extraction methods, and the part of the plant used (Do et al. 2015). Yet, solubility and stability issues with essential oils have prompted advances in delivery systems, notably emulsions and nanoemulsions, which are expected to enhance the effectiveness of these natural compounds.

Emulsions are colloidal systems in which two immiscible liquids, such as oil and water, are mixed with the aid of a surfactant that stabilizes the blend (Behjati et al. 2017). Nanoemulsions, a subtype of emulsions, are nanometric-sized mixtures that retain these characteristics but with small droplet diameters, typically ranging from 20 to 200 nm (Kumar et al. 2019; Mahdi and Maraie 2019). They are formulated by mixing at least three components: oil, water, and an emulsifier. The preparation methods employed in nanoemulsion formulation are broadly classified into two principal categories: high-energy and low-energy, based on the energy requirements, the type of phase inversion, and the capacity for self-emulsification. These techniques are critical, significantly influencing their physical stability, bioavailability, and therapeutic efficacy.

The present study, therefore, assesses the insecticidal efficacy of sweet orange essential oil and d-limonene as well as their emulsions and high-energy and low-energy nanoemulsions against *D. suzukii*. Several essential oils, such as those derived from citrus species, are classified as GRAS (Generally Regarded as Safe) by the United States Food and Drug Administration (FDA) due to their favorable safety profiles (Manzur et al. 2023). Brazil stands out as the largest global producer of citrus fruits, producing 585,448 hectares in May 2022/23 (IBGE 2024), increasing the availability of citrus EO for use in the agricultural and food industries. Moreover, different previous studies have shown that EOs from the peel of *Citrus* spp. exhibit insecticidal activities against different insects, such as houseflies, mosquitoes, stored product weevils, cotton aphids, lepidopteran caterpillars, and fruit flies (Giunti et al. 2018, 2019; El Kasimi et al. 2023; Manzur et al. 2023; Murugan et al. 2012; Oyedeji et al. 2020; Usseglio et al. 2022). Subsequently, the study examines the impact of the different emulsified solutions of sweet orange essential oil and d-limonene on the non-target organism, *Pachycrepoideus vindemmiae* (Rondani)(Hymenoptera: Pteromalidae). This generalist wasp is reported as a parasitoid of the pupal stage of various Drosophilidae fly families (Yang et al. 2019), including *D. suzukii* (Mariano-Macedo et al. 2020), and has been introduced for classical biological control programs targeting fruit flies in different Latin American countries.

## Materials and methods

### Essential oil and d-limonene specifications

The *Citrus sinensis* essential oil (SCEO) was purchased from WNF essential oils Indústria e Comercio Ltda (São Paulo, Brazil). According to the manufacturer, the EO is obtained by either cold pressing or hydrodistillation of sweet orange fresh peels. The chemical characterization of the *C. sinensis* essential oil was carried out and revealed that its chemical composition included six monoterpenes, with limonene being the major component (95.29%), followed by β-pinene (1.29%), γ-terpinene (1.22%), p-cymene (0.94%), myrcene (0.75%), and α-pinene (0.51%) (Pineda et al. 2025). The (R)-(+)-d-limonene (97 % purity) was purchased from Sigma-Aldrich (Jurubatuba, SP). Tween 80 was purchased from Dinâmica Química Contemporânea Ltda (Indaiatuba, SP).

### Rearing of *Drosophila suzukii* flies and *Pachycrepoideus vindemmiae* parasitoids

Rearing of spotted wing dorosphila *D. suzukii* and the pupal parasitoid *P. vindemmiae* were maintained at the Molecular Entomology and Ecotoxicology Laboratory (MEET), Entomology Department, Federal University of Lavras. The adults, larvae, and pupae of *D. suzukii* were housed in transparent plastic cages (V = 700 mL, 4.7 cm in height × 6.7 cm in diameter) sealed with voile mesh under controlled insectary conditions (24 ± 2 °C; 60 ± 5% and photoperiod: 12:12 h L/D). They were fed an essential cornmeal diet of cereal flour, corn flour, and water, supplemented with dried yeast. Nipagin was added as an antifungal agent (Andreazza et al. 2016; Pineda et al. 2023).

Colonies of the parasitoid *P. vindemmiae* were maintained in a climate-controlled chamber at 26 ± 1 °C, with 60 ± 5 % relative humidity and a 12 h:12 h photoperiod and were housed in 1.8-liter plastic containers supplied with streaks of pure honey. One to two day-old pupae of *Mosca domestica*, affixed to a 7x5 cm^2^ piece of cardboard using non-toxic white glue, were used as hosts for the parasitoid’s reproduction. The pupae were exposed to the parasitoids for 48 h under controlled environmental conditions. After this period, the parasitized pupae were carefully removed, transferred to clean containers, where they were kept for approximately 17 days for the emergence of new adult wasps (Farro-Barbarán et al. 2026).

### Essential oil and d-limonene solutions preparation

Based on preliminary tests, five to eight concentrations, causing mortalities between 0 and 100%, were used for each formulation to determine the concentration-mortality curves and the lethal concentrations (LC_50_ and LC_90_) that were subsequently used in different bioassays. Thus, the non-emulsified orange essential oil solutions (SCEO) were prepared at concentrations of 1, 1.5, 2, 2.5, 2.75, 3, and 3.5 percent by weight (wt%), while for d-limonene, the solutions were prepared at concentrations of 0.25, 0.5, 1, 1.5, and 2 wt%. Each of the prepared solutions was solubilized in Tween^®^ 80 (0.05 wt%) and distilled water, also used as controls. Before pipetting, the solutions were vigorously vortexed for 10 s to ensure a thorough dispersion of the oil in the water.

#### Preparation of orange essential oil and d-limonene emulsions

The emulsions of the SCEO and d-limonene were prepared by gradually and continuously adding the EO and surfactant to water, followed by magnetic stirring at 750 rpm. Varying concentrations of EO (1.0, 1.25, 2.0, 2.25, 2.5, 3.5, 4.0, and 5.0 wt%) and of d-limonene (0.5, 2.0, 2.5, 3.5, 4, and 5.0 wt%) were used to prepare the oil phase for the emulsion formulation. The surfactant-to-oil ratio was fixed for all emulsions at 3:1.

#### Preparation of orange essential oil and d-limonene low-energy nanoemulsions

Nanoemulsions were formed using a method based on spontaneous emulsification, as described by Chang et al. (2013), with minor modifications. Standardized conditions for the experiments were as follows: 80 wt% citrate buffer system (5 mM, pH 3.5), 10 wt% Tween 80, and 10 wt% total oil phase. The oil phase was prepared by mixing medium-chain triglycerides (MCT) with the essential oil at various mass contents (SCEO at 2.5, 2.75, 3, 3.5, 5, and 5.5 wt%, and d-limonene at 1.5, 2.5, 3, 3.5, 5, and 7 wt%). This mixture was then added to an aqueous phase containing citrate buffer with Tween 80 and stirred magnetically at 750 rpm at ambient temperature. The emulsions were subsequently homogenized at 9500 rpm for 4 minutes using an IKA^®^ T10 basic ULTRA-TURRAX (IKA^®^ SP, Brazil). Subsequently, the obtained emulsions were protected from light and stored at 4 °C.

#### Preparation of orange essential oil and d-limonene high-energy nanoemulsion

A two-step process was employed to prepare the oil-in-water (O/W) nanoemulsions. SCEO and d-limonene emulsions were prepared using the same protocol employed for low-energy nanoemulsions as described previously. After homogenization at 9500 rpm, the emulsions were sonicated to obtain smaller droplet sizes and a more uniform particle size distribution. For that, an ultrasonic cell crusher (Model QR200, 200W, Eco sonics—Ultronique, Sao Paulo, Brazil) was used to ultrasonicate the emulsions for ten minutes (frequency of 20 kHz; sonication amplitude of 50% and sonication power of 100 W). To control the temperature (and thus isolate its effects) during the ultrasonication process, all samples were kept in an ice bath. After sonication, the samples were protected from light and stored at 4 °C.

### Nanoemulsion characterization

The droplet sizes, polydispersity index (PDI), and zeta potential of CSEO and d-limonene nanoemulsions were measured at 25 °C based on the zeta potential/particle size analyzer of DLS technology using Zetasizer Nano ZS (ZEN3600, Malvern Instruments, Brazil) at the Empresa Brasileira de Pesquisa Agropecuária (EMBRAPA) de Instrumentação (São Carlos, SP. These measurements were conducted explicitly at the nanoemulsions’ lethal concentration levels, CL_50_ and CL_90_. All samples were tested in triplicate. The viscosity for the prepared nanoemulsion was measured in a Brookfield digital Viscometer with the spindle no. 63. Since viscosity could be measured as a function of temperature, the experiment was performed at ambient room temperature (25 ± 2 °C) for all the samples. Furthermore, the prepared nanoemulsions were kept in screw-capped test tubes at 4 °C for 7 days to evaluate their physical stability, including assessments of their size, strength, and viscosity.

### Toxicity bioassays of pure, emulsified, and nanoemulsified *Citrus sinensis* EO and d-limonene with the spotted wing drosophila flies

#### Toxicity to *Drosophila suzukii* adult flies

The toxicity assessment of SCEO, d-limonene, their emulsions, and nanoemulsions against *D. suzukii* followed a bioassay based on the IRAC protocol No. 26, with minor adjustments (Pineda et al. 2023). Twenty-five adult *D. suzukii* individuals aged 3–5 days were introduced into 200 mL glass flasks containing a dental cotton roll (Size #2, 1–1/2″ × 3/8″) impregnated with 2.2 mL of each treatment. The flasks were sealed with foam plugs to prevent any potential escape of flies. After the insertion of the emulsion-impregnated cotton roll, 25 non-sexed flies were introduced into individual glass flasks (replicate) for 24 h, following which mortality rates were evaluated. Five to seven concentrations were used for each treatment, and all concentrations were replicated (one flask per replicate) four times, and were maintained under controlled conditions (T: 23 ± 2 °C, RH: 60 ± 5%, and photophase of 12H). Distilled water, Tween, and the buffer used to formulate the emulsions were considered as controls in all toxicity bioassays.

### Toxicity to *Drosophila suzukii* larvae and pupae

The lethal concentrations (LC_50_) determined in the previous tests with adults for each preparation (pure, emulsion, and nanoemulsion) (see Supplementary Table 1 in the results section) were used to assess the larvicidal and pupicidal activities against the third instar larvae and one-day-old pupae of *D. suzukii*.

Third-instar larvae were gently dipped for 10 s into the different solutions of pure, emulsified, and nanoemulsified orange EO and d-limonene. Subsequently, the larvae were transferred to Petri dishes (30 mm in diameter × 15 mm in height) containing artificial diet. Each treatment (pure, emulsified, and nanoemulsified EO and d-limonene and the controls) was tested in four replicates, with each replicate consisting of ten larvae. Larval mortality was assessed at 24 h by touching each larva with a paintbrush, and those not responding were considered dead. The progression of the surviving larvae into pupae and subsequently into adults was monitored up to 10 days after application, considering that the developmental period from egg to adult for *D. suzukii* is approximately 9–10 days at a constant temperature of 25 °C (Winkler et al. 2020).

The experiment with pupae was carried out as described above for the larvae with ten (10) one-day-old pupae for each treatment. The pupal mortality was subsequently assessed by counting those pupae from which no adults emerged within 10 days. Individuals displaying visible morphological alterations, such as unsuccessful emergence and deformities in the abdomen, wings, legs, and pronotum, were classified as deformed adults.

### Toxicity bioassays of pure, emulsified, and nanoemulsified *Citrus sinensis* EO and d-limonene with the parasitoid *Pachycrepoideus vindemmiae*

#### Toxicity to the adults of the parasitoid *Pachycrepoideus vindemmiae*

To determine the toxicity of the CSEO, d-limonene, and their nanoformulations to *P. vindemmiae*, the LC_50_ and LC_90_ concentrations determined in the toxicity bioassays with the adult flies were employed. A volume of 200 µL of each formulation was applied to a piece of Whatman filter paper (No. 1) (20 mm diameter), which was then placed at the bottom of a flat-bottomed glass tube (height = 18.5 cm and diameter = 2 cm). Then, ten adult wasps, three to five day-old, were introduced into each test tube, and the tubes were sealed with foam plugs to prevent escape. Parasitoid mortality was assessed 24 h after exposure initiation. The experimental design was completely randomized, with 10 replicates per treatment conducted under controlled conditions (temperature: 27 ± 2 °C, relative humidity: 60 ± 5%, and a 12-h photoperiod).

#### Effects on the parasitism activity of *Pachycrepoideus vindemmiae* females

The potential impact of CSEO, d-limonene, and their nanoformulations on the parasitism activity of *P. vindemmiae* female wasps on *D. suzukii* pupae was assessed. Each experimental unit consisted of a glass test tube with 15 recently formed (within a maximum of 24 h) *D. suzukii* pupae. These pupae, previously dipped in each treatment for 10 s, were subsequently attached to a 1.5 × 2.5 cm piece of cardboard using non-toxic white glue. The pupae were exposed to three to four-day-old adult wasp females for 24 h. Each treatment included ten repetitions, and the bioassay was carried out under controlled laboratory conditions, maintained at 26 ± 1 °C, 60 ± 5 % humidity, and 12 h:12 h photoperiod. The emergence of wasps and SWD flies was monitored until the 22^nd^ day after the start of the experiment.

### Statistical analyses

The concentration-mortality data of *D. suzukii* adult flies were subjected to probit analysis (SAS Institute, Cary, NC, USA). Toxicity data for the *D. suzukii* larvae and pupae, for the adults of *P. vindemmiae,* and the parasitism of the wasp females were analyzed using one-way ANOVA when assumptions were met and ANOVA on Ranks (Kruskal-Wallis test) when data deviated from normality, ensuring the appropriate statistical approach for each dataset. Pairwise multiple comparisons were conducted using either the Holm-Sidak, Tukey, or Dunn’s post hoc tests. Statistical analyses were conducted using R software (version 2022.12.0).

## Results

### Characterization of the prepared low and high-energy nanoemulsions

The physical characterization of the prepared low and high-energy nanoemulsions of CSEO and d-limonene was carried out by assessing their size, polydispersity (PDI), zeta potential, and viscosity (Table 1). Overall, the globule size and polydispersity were higher in the low-energy (617.36–796.46 nm; PDI: 0.548–0.644) nanoemulsions compared to the high-energy (221.26–289.7 nm; PDI: 0.210–0.255) ones. Moreover, the zeta potential values of low and high-energy nanoemulsions were within the narrow range of − 2 to − 7, and their viscosity ranged at ambient room temperature (25 °C) within 2.0–3.5 cP.

**Table 1:** Physical parameters of the prepared nanoemulsions at day 1 and after 7 days of storage at 4 °C

Time	TRT	Lethal concentration	Average globule size (nm)	Standard error	PDI	Zeta potential (mV)	Viscosity (cP) T 25 °C
Day 1	*C. sinensis* low Energy	LC_50_	796.46	0.075	0.633	− 2.00	3.48
		LC_90_	617.36	0.262	0.548	− 1.38	3.65
	Limonene low Energy	LC_50_	768.43	0.068	0.644	− 1.89	3.65
		LC_90_	693.10	0.258	0.594	− 1.34	3.72
	*C. sinensis* high energy	LC_50_	225.40	0.021	0.217	− 3.25	2.69
		LC_90_	289.70	0.025	0.210	− 6.77	3.68
	Limonene high energy	LC_50_	221.26	0.008	0.226	− 3.89	3.27
		LC_90_	256.33	0.015	0.255	− 1.92	2.89
Day 7	*C. sinensis* low Energy	LC_50_	3985.0	0.462	0.948	− 1.23	2.79
		LC_90_	3952.0	3.291	0.781	− 0.86	3.09
	Limonene low Energy	LC_50_	3406.8	4.911	1.000	− 1.15	3.06
		LC_90_	1740.1	2.970	0.739	− 1.08	3.24
	*C. sinensis* high energy	LC_50_	289.33	0.057	0.273	− 2.82	2.05
		LC_90_	354.56	0.078	0.377	− 5.90	2.85
	Limonene high energy	LC_50_	314.90	0.182	0.282	− 3.27	3.09
		LC_90_	309.30	0.017	0.236	− 1.56	2.76

After 7 days of storage at 4 °C, both low and high-energy nanoemulsions demonstrated an increase in droplet size, polydispersity index (PDI), and viscosity, while the zeta potential values remained relatively stable across formulations (Table 1).

### Susceptibility of adult *Drosopihla suzukii* exposed to different preparations of CSEO and d-limonene

**Fig. 1:**
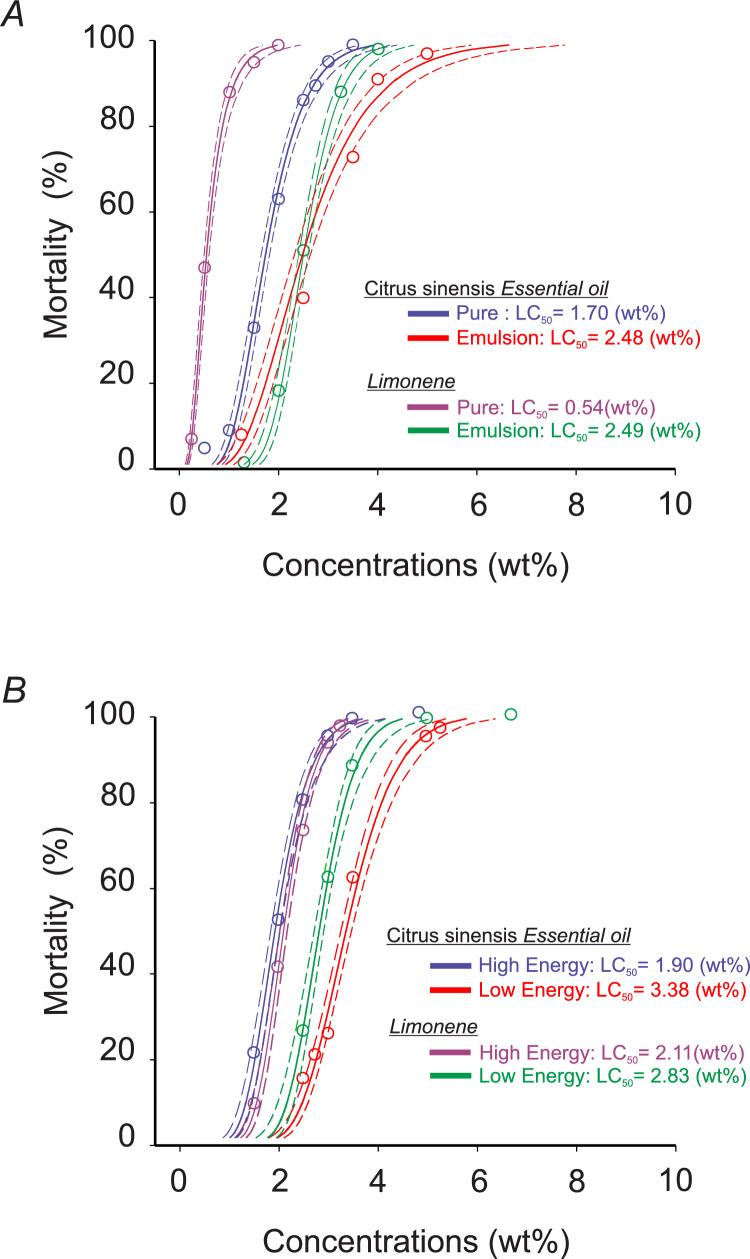
Toxicity curves of pure, emulsified solutions (**A**), high-energy and low-energy nanoemulsions (**B**) of *Citrus sinensis* essential oil and d-limonene formulations to *Drosophila suzukii* flies

The different concentrations-mortality curves of the SCEO, d-limonene, their emulsions, and nanoemulsions were estimated for adult *D. suzukii* after 24 h of exposure using Probit analysis (Figure 1 and supplementary Table 1). The most toxic preparations were pure CSEO (CL_50_= 1.7 wt%, CL_90_= 2.72 wt%, χ^2^= 2.50, df = 5, *P* = 0.77) and pure d-limonene (CL_50_= 0.54 wt%, CL_90_= 1.11 wt%, χ^2^= 1.76, df = 3, *P* = 0.62) (Figure 1A). These were followed by high-energy nanoemulsion of CSEO (HE. CSEO) with CL_50_= 1.90 wt%, CL_90_= 2.77 wt% (χ^2^ = 1.4, df = 4, *P* = 0.84) and high-energy nanoemulsion of d-limonene (HE. LIM) with CL_50_= 2.11 wt%, CL_90_= 2.84 wt% (χ^2^ = 1.60, df = 4, *P* = 0.80) (Figure 1B). The emulsion of CSEO (E. CSEO) showed CL_50_= 2.48 wt%, CL_90_= 4.26 wt% (χ^2^= 6.0, df = 4, *P* = 0.19), and the emulsion of d-limonene (E.LIM) had CL_50_= 2.49 wt% and CL_90_= 3.34 wt% (χ^2^= 4.6, df = 4, *P* = 0.20). The preparations with the lowest toxicity were the low-energy nanoemulsion of CSEO (LE. CSEO) with CL_50_ = 3.38 wt%, CL_90_ = 4.56 wt%, (χ^2^ = 3.79, df = 4, *P* = 0.43), and the low-energy nanoemulsion of d-limonene (LE.LIM) with CL_50_ = 2.83 wt%, CL_90_ = 3.67 wt% (χ^2^ = 3.20, df = 3, *P* = 0.36). The control group showed a mortality rate lower than 2%.

### Toxicity of different preparations of CSEO and d-limonene to *D. suzukii* larvae and pupae

**Fig. 2:**
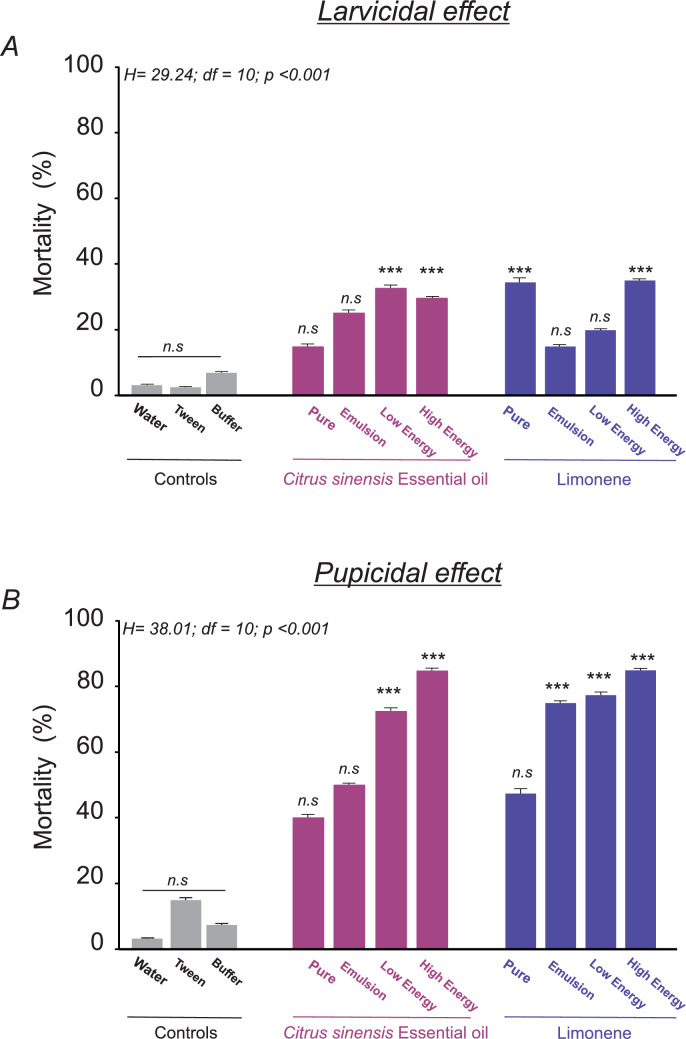
Larvicidal (**A**) and pupicidal (**B**) activities of different *Citrus sinensis* essential oil and d-limonene formulations. *n.s.* indicate no significant differences, and (***) indicate significant differences at *P* < 0.001 (ANOVA on Ranks). The data are reported as means ± SE, and each bar represents the mean of four replicates per treatment with 10 individuals (larvae or pupae) per replicate

Total accumulated mortality, encompassing dead larvae and non-emergent pupae, was evaluated and presented as a percentage of mortality (Figure 2A). Overall, the mortality of exposed larvae did not exceed 40% in any of the treatments. Pure d-limonene and high-energy nanoemulsion of d-limonene (HE. LIM) demonstrated the highest efficacy, achieving a mortality rate of 35%. The low-energy nanoemulsion of CSEO (LE. CSEO) also showed high efficacy, with an average mortality of 32.5%. The high-energy nanoemulsion of CSEO (HE. CSEO) followed closely, exhibiting a mortality rate of 30%. Emulsions of CSEO and d-limonene (E. CSEO and E. LIM) presented average mortalities of 25% and 15%, respectively. Treatments with low-energy nanoemulsion of d-limonene (LE. LIM) and pure CSEO exhibited average mortalities of 20% and 15%, respectively. The additional controls, consisting of Tween and buffer, showed low mortality rates of 2.5% and 7.5%, respectively, similar to those observed in the control group. These findings confirm that the observed toxic effects are primarily attributable to the CSEO and d-limonene rather than the other solution’s components.

Different from the larval results, the mortality of exposed pupae was higher than 40% for all the treatments with statistically significant differences (H = 38.014, df = 10, *P* < 0.001) between treatment groups and compared to the controls (Figure 2B). Notably, the high-energy nanoemulsions of d-limonene (HE. LIM) and CSEO (HE. CSEO) were the most effective, with mortality rates of 85% in both cases (*P* = 0.001). Other formulations, including low-energy nanoemulsions of d-limonene (LE. LIM) and CSEO (LE. CSEO), as well as the emulsion of d-limonene (E. LIM), also exhibited significantly higher mortality rates, achieving 77.5%, 72%, and 75%, respectively. Other preparations, including CSEO emulsion (E. SCEO), pure d-limonene, and pure CSEO essential oil, showed moderate mortality with 50%, 47.5%, and 40% mortality rates, respectively (Figure 2B).

In addition to assessing direct pupal mortality, we examined the occurrence of morphological malformations in *D. suzukii* adults completely or partially emerged from exposed pupae (Supplementary Figure 1). Significant differences in the number of emerged adults with malformations were found between the treatments groups (H = 25.276, df = 10, *P* = 0.005). In particular, emulsified formulations of CSEO (E.CSEO) and d-limonene (E. LIM) and low-energy nanoemulsion of CSEO (LE. CSEO) caused pronounced effects with E.CSEO and LE. CSEO each showing a 30% of malformations incidence, while E. LIM had an incidence of 27.5%, followed by high-energy nanoemulsion of CSEO (HE. CSEO) with 17.5%, being largely different from the control (less than 4% of mortality and malformations).

### Toxicity to *Pachycrepoideus vindemmiae* and effect of different preparations of CSEO and d-limonene on its parasitism activity

**Fig. 3:**
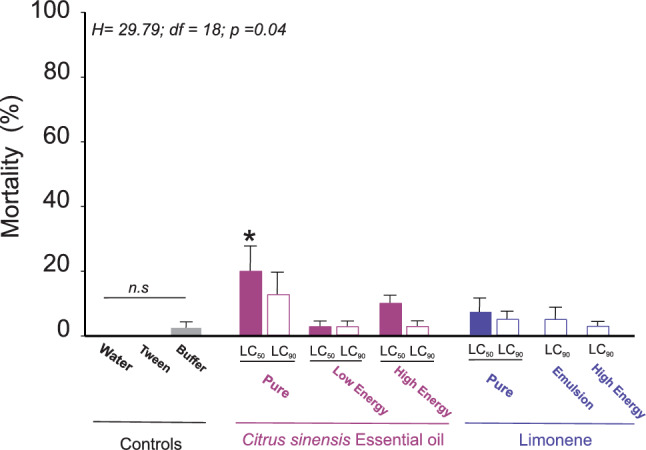
Mortality rates induced in adult females of the parasitoid wasp *Pachycrepoideus vindemmiae* by the LC_50_ and LC_90_ lethal concentrations of emulsified solutions and high-energy and low-energy nanoemulsions of *Citrus sinensis* essential oil and d-limonene formulations, determined for *Drosophila suzukii* adult flies. Only the treatments that caused mortalities different from zero are represented. *n.s.* indicates no significant differences, and (*) indicates significant differences at *P* < 0.05 (ANOVA on Ranks). The data are reported as means ± SE, and each bar represents the mean of 10 replicates per treatment with 10 female wasps per replicate

As for the toxicity of the different preparations of CSEO and d-limonene on *P. vindemmiae* after 24 h of exposure, no high mortality was observed for different treatments at both LC_50_, LC_90_ concentrations, except the LC_50_ of the orange pure EO which showed a mortality of 20% being statistically different (H = 29.793, df = 18, *P* = 0.040) from the other preparations (Figure 3).

The potential impact of CSEO, d-limonene, and their nanoformulations on the parasitism activity of *P. vindemmiae* female wasps was assessed by exposing them to treated pupae of *D. suzukii*. Generally, all treatments at their LC_50_ and/or LC_90_ concentrations either slightly reduced or had no impact on the *P. vindemmiae* parasitism (H = 118.260, df = 18, *P* < 0.001) (Table 2 and Supplementary Table 2). The LC_50_ concentrations of all treatments decreased *P. vindemmiae* parasitism, with parasitism rates ranging from 66% (CSEO) to 78.33% (LE-CSEO), except for *C. sinensis* High Energy nanoemulsions (91.33%), which was similar to the controls. A similar decrease trend was observed for the LC_90_ concentrations except for the d-limonene (80%) that showed no statically differences (*P* > 0.05) with the control.

**Table 2:** Effect of different preparations of *Citrus sinensis* essential oil and d-limonene on the parasitism of *Pachycrepoideus vindemmiae*

TRT	Lethal concentration	Parasitized^a^	Parasitism (%)^b^
Negative control	–	0	0
Positive control	–	147	98.33
Tween	–	148	98.66
Buffer	–	148	98.66
*C. sinensis* EO (pure)	LC_50_	99	66*
	LC_90_	105	70*
Limonene (pure)	LC_50_	103	68.66*
	LC_90_	120	80
*C. sinensis* emulsion	LC_50_	108	72*
	LC_90_	113	75.33*
Limonene emulsion	LC_50_	109	72.66*
	LC_90_	118	78.66
*C. sinensis* low energy	LC_50_	118	78.33
	LC_90_	100	66.66*
Limonene low energy	LC_50_	116	77.33*
	LC_90_	117	78*
*C. sinensis* high energy	LC_50_	137	91.33
	LC_90_	105	70*
Limonene high energy	LC_50_	112	74.66*
	LC_90_	101	67.33*

*Indicate a significant statistical difference using ANOVA on Ranks (Kruskal–Wallis test) and Dunn’s (*P* < 0.05) as a post-hoc test

^a^Indicates fly’s pupae parasitized by *P. v*indemmiae and presenting an emergence hole, or fly’s pupae with the entire parasitoid individual visible inside puparium shell under a microscope

^b^Parasitism rate estimated as % parasitized pupae (N = 150).

## Discussion

Recently, botanical extracts as essential oils have been investigated as alternatives to mitigate environmental and health problems caused by the widespread use of synthetic pesticides. However, despite the large body of literature reporting the efficacy of different EOs under laboratory conditions, their use as biopesticides has been facing many challenges deriving from their biochemical characteristics, leading to an inherent instability under environmental conditions. In this context, innovative formulations based on nanotechnologies have been explored. In the present study, we prepared emulsions, high and low energy nanoemulsions of sweet orange essential oil and its major components, and tested their stability, toxicity to different stages of the spotted wing drosophila, and their selectivity to the pupal parasitoid *P. vindemmiae.* Our results revealed that the emulsion processes, besides having a key role in the size and stability over time of the prepared particles, greatly impact the bioactivity of the sweet orange essential oil and its major component (d-limonene) as well as its selectivity to non-target organisms and the services they provide.

Nanoemulsions are generated using either high-energy or low-energy emulsification methods (Sarmah et al. 2025). Here, we prepared tween-water emulsions of orange essential oil and d-limonene, which we subsequently used to produce low-energy and high-energy nanoemulsions. The physical characterization revealed significant differences between the prepared nanoemulsions, confirming that the efficiency of producing nanoemulsions and their characteristics can vary significantly depending on the type of surfactant used and the experimental conditions, such as temperature and the synergy between the components of the surfactant/co-surfactant system (Silva et al. 2015; Pavoni et al. 2020; Campolo et al. 2020). Indeed, the nanoemulsions obtained by high energy showed droplet sizes between 200 and 300 nm, with a polydispersity index (PDI) of less than 0.3, indicating a homogeneous dispersion and potential for physical stability. In contrast, those produced by the low-energy method exhibited larger particle sizes (600 and 800 nm) and higher PDI (0.5–0.65), suggesting a potentially lower stability. This potential difference in the stability was evidenced by measurements taken after 7 days of storage at 4 °C, which showed a notable increase in droplet size in all formulations, being more pronounced in the nanoemulsions obtained by the low-energy method. Lower PDIs of nanoemulsions were previously reported to support their long-term stability, resulting in minimal changes in droplet size, even after two months of storage (Singh and Pulikkal 2022). In addition, the results indicated that, based on the size of the particles, the emulsions obtained fall more appropriately into the category of microemulsions. Furthermore, the zeta potential in the range of − 2 to – 7 mV implies low colloidal stability. While these values suggest some electrostatic stability, they are relatively low and it may not be enough for preventing particle aggregation. Values further from zero increase the particle repulsion, thus avoiding aggregation and improving emulsification stability (de Souza et al. 2025a). The process of Ostwald ripening, in which the growth of larger droplets at the expense of smaller ones occurs, is a reason for this observed trend of instability conducted on low-energy nanoemulsions, which could lead to a polydispersity increase and potential loss of system stability (Reyes et al. 2021). These results further emphasize the importance of optimizing the formulation and storage conditions of nanoemulsions to improve their stability along with long-term functionality for industrial use.

The toxicity assessment of various preparations of CSEO and d-limonene on *D. suzukii* adult flies highlights the significant influence of the formulation method on insecticidal effectiveness. Although the pure essential oil proved to be more toxic, with lower lethal concentrations, the differences in toxicity between the pure CSEO oil and high-energy nanoemulsions were not substantially large. Such a good performance of the nanoemulsions can be attributed to their smaller size allowing them to easily penetrate the insect’s cuticle and even individual cells, where they can interfere with physiological processes. It is worth highlighting that in the present study, unsexed adult flies were used to simulate natural conditions but an assessment of the prepared nanoemulsions in separated sexes will give a better understanding on their activities especially that males of *D. suzukii* are reported to present generally higher susceptibility to insecticides than the females (de Souza et al. 2022a, b; de Souza et al. 2024; Kim et al. 2016; Park et al. 2016). Furthermore, High-energy nanoemulsions showed lower LC_50_ and LC_90_ values compared to low-energy nanoemulsions and the emulsions. The findings underscore how high-energy methods for preparing nanoemulsions enhance their stability, bioavailability, and controlled release towards *D. suzukii*. This better activity may be due to the production of finer and more uniformly distributed particles, which facilitate better coverage and penetration into the insect tissues, increasing the bioavailability of the active ingredients (Gupta et al. 2024; Zahi et al. 2015). High-energy emulsification processes were reported to significantly increase the residual toxicity of garlic EO against 3rd instar *Planococcus citri* nymphs, reinforcing the relevance of high-energy nanoemulsification for enhancing insecticidal performance (Modafferi et al. 2025). Given the advantages of nanoemulsions in terms of application and potential long-term stability, the toxicity results reinforce that high-energy nanoemulsification can be considered a viable alternative that does not significantly compromise efficacy.

On the other hand, following exposure to LC_50_ concentrations, *D. suzukii* pupae exhibited markedly higher susceptibility to CSEO and d-limonene preparations than exposed larvae. Actually, pupal mortality was substantially higher, reaching 85% for d-limonene (HE. LIM) and CSEO (HE. CSEO) nanoemulsions, while low-energy formulations produced mortality rates between 75 and 77.5%, and pure essential oil preparations showed lower toxicity in pupae, with mortality values of 40–45%. In contrast, in larvae, mortality rates remained relatively low, ranging from 30 to 35%, with the highest efficacy observed for pure d-limonene and high-energy nanoemulsion of d-limonene (HE-LIM). The differences in the susceptibilities of larvae and pupae might derive from the significant differences in the metabolic processes of these two stages. During the pupation phase, the Malpighian tubules, essential for rapidly eliminating waste and toxic solutes, stop their physiological activity, as evidenced by the loss of apical microvilli (Cohen et al. 2020). This inactivity can make pupae more susceptible to the action of nanoemulsions. The differences in the effects caused by produced nanoemulsions in different life stages emphasize the need for nano-specific and standardized exposure and toxicological testing methods and protocols.

In addition to the toxicity of the various preparations studied on *D. suzukii*, it is also necessary to consider their effect on natural enemies and non-target organisms (Eben et al. 2020; Souza et al. 2022a, b; Gowton et al. 2020). All the preparations analyzed caused low mortality in adults of *P. vindemmiae*, although a reduction in parasitism rates was also observed. These results suggest that the produced formulations have potential as alternatives to Integrated Pest Management (IPM) of *D. suzukii* (Eben et al. 2020; Souza et al. 2022a, b) but also underscore the importance of studying the potential impacts of plant-based biopesticides and nanoformulations on different parasitoid species and other non-target organisms on a case-by-case basis, as these interactions can vary significantly (Sombra et al. 2022; Haddi et al. 2020; van Oudenhove et al. 2023). In this regard, the effects of plants secondary metabolites in their pure and nanoformulated forms were evaluated against adults of *D. suzukii*, as well as on the survival and reproductive parameters of non-target organisms, observing that terpenes and phenylpropanoids, both in their pure and nanoformulated forms, did not affect parasitism, emergence, or sex ratio of *Palmistichus elaeisis* (de Souza et al. 2025a) and the survival and feeding capacity of the non-target organism the earwig *Doru luteipes* were not significantly affected by exposure to the selected terpenes and phenylpropanoids (de Souza et al. 2024). Furthermore, Chitosan-sodium alginate nanocapsules carrying EOs of *Piper marginatum* caused mortality rates above 90% to *D. suzukii* adults but with low mortality and with no interference in parasitism by surviving females of its parasitoid *Trichopria anastrephae* (de Souza et al. 2025b). Similarly, different nanoformulations of plant-based extracts have been tested for their potential insecticidal activity and selectivity to non-target organisms. In this context, the insecticidal efficacy of garlic EO-based nanoemulsions exhibited high toxicity (100% mortality within 24 h) to the invasive citrus pest *Delottococcus aberiae*, while demonstrating no toxicity (100% of survival) toward predatory beetle *Cryptolaemus montrouzieri* (Modafferi et al. 2025). Additionally, microcapsules of esculetin, a principal compound in *Albizia kalkora* tested against *Hyphantria cunea*, had negligible adverse effects on *Danio rerio* (Cyprinidae: Cypriniformes) and *Arma chinensis* (Hemiptera: Pentatomidae) (Yue et al. 2025), and nanoemulsions of *Salvia officinalis* essential oil and one of its major components, camphor, tested as eco-friendly natural green formulations against *Sitophilus oryzae*, were reported safe toward *Aporrectodea caliginosa* earthworms at concentrations up to 150 mg kg−1 soil (Albogami et al. 2026).

The present study highlights the complexity and importance of choosing methods for emulsifying and formulating essential oils for insecticide applications. Nanoemulsions prepared by high-energy techniques demonstrated greater efficacy, stability, and uniformity in particle distribution compared to low-energy preparations, which, although less effective, remain a viable alternative, especially in scenarios where cost and simplicity of production are critical factors. The toxicity of the formulations at different stages of *D. suzukii* development, combined with the reduced impacts on natural enemies such as *P. vindemmiae*, indicates that these essential oil nanoformulations could be a promising and environmentally sustainable tool once the long-tern stability challenges are been overcome.

## Supplementary Information

Below is the link to the electronic supplementary material.

Supplementary file1 (PDF 385 KB)

Supplementary file2 (DOCX 18 KB)

## Data Availability

The datasets generated and/or analyzed during the current study are available from the corresponding author upon reasonable request.
