# Supplementary material for: Toxicity of emulsions, high and low energy nanoemulsions of orange essential oil and d-limonene to Drosophila suzukii, and selectivity to Pachycrepoideus vindemmiae
Source: 3 Biotech. 2026 May 14;16(6):191. doi: 10.1007/s13205-026-04833-9 (PMC13172244; doi:10.1007/s13205-026-04833-9)

Supplementary Figure 1. Normal feature and developmental anomalies observed in *D. suzukii* pupae and adults. A) Pupa undergoing normal development with visible distinguishable features such as developing appendages and characteristic pigmentation.(B): pupa with arrested development and lacking clear morphological differentiation features. (C): partial emergence, where the adult flies failed to exit completely, leading to death. (D): varying degrees of emergence failure, indicating a potential defect during the emergence process. (E) An adult fly with deformations in the cephalic and thoracic exoskeletons and wings. (F): An individual with pupal emergence failure shows and another adult with cephalic and thoracic malformations, as well as disorganization of the appendages.

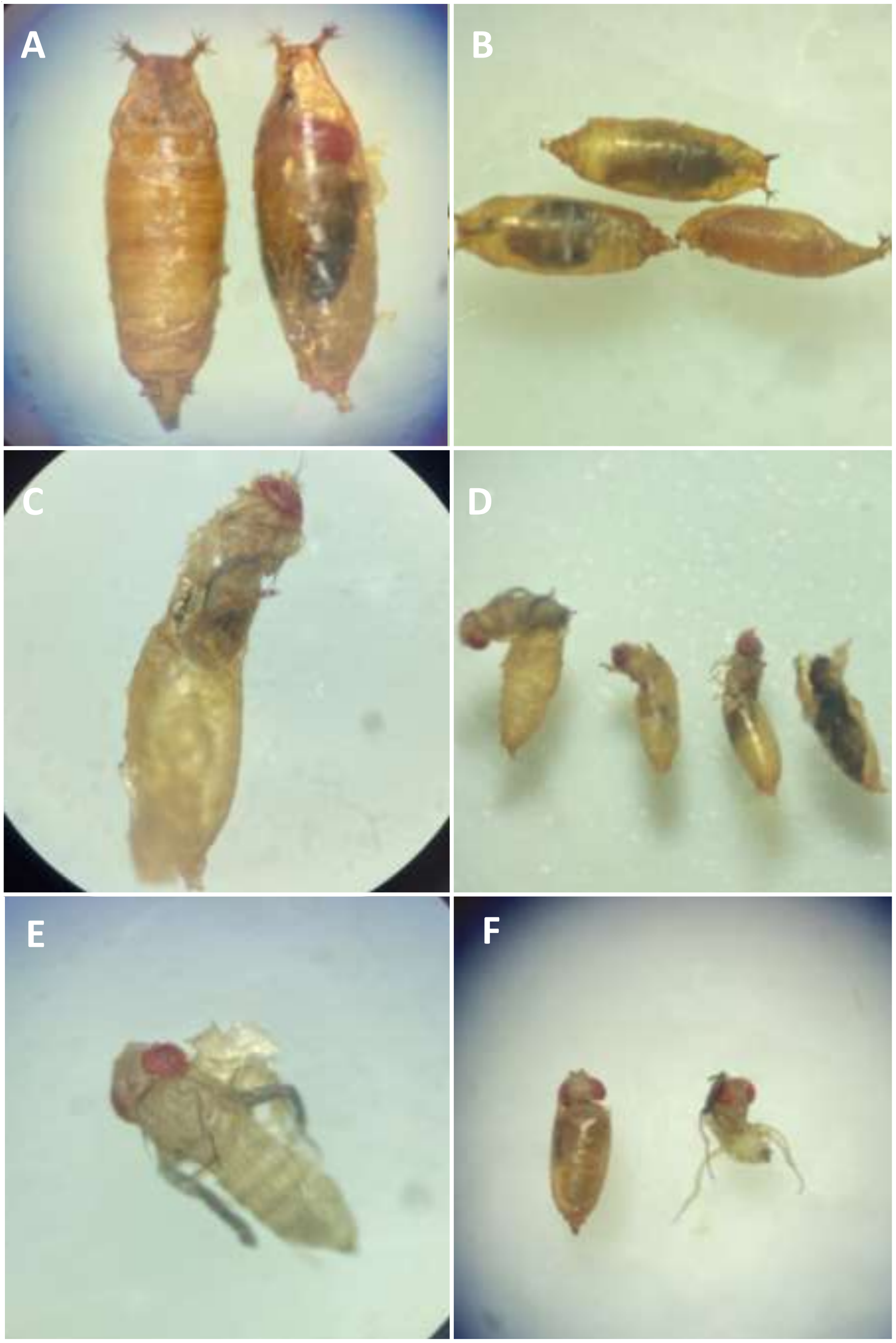

Supplement: Supplementary file 1 — Supplementary file1 (PDF 385 KB) [file 13205_2026_4833_MOESM1_ESM.pdf]
