# Supplementary material for: Toxicity of emulsions, high and low energy nanoemulsions of orange essential oil and d-limonene to Drosophila suzukii, and selectivity to Pachycrepoideus vindemmiae
Source: 3 Biotech. 2026 May 14;16(6):191. doi: 10.1007/s13205-026-04833-9 (PMC13172244; doi:10.1007/s13205-026-04833-9)
Supplement: Supplementary file 2 — Supplementary file2 (DOCX 18 KB) [file 13205_2026_4833_MOESM2_ESM.docx]

**Supplementary Table 1:** lethal concentrations LC₅₀ and LC₉₀ of the *Citrus sinensis* essential oil, D-limonene, their emulsions, and high and low-energy nanoemulsions against *D. suzukii* adults

|  | **TRT** | | **Nº insect** | | **LC_50_ (F.I)** | | **LC_90_ ( F.I)** | | **χ^2^** | | ***df*** | | ***p*** | |  |
| --- | --- | --- | --- | --- | --- | --- | --- | --- | --- | --- | --- | --- | --- | --- | --- |
| ***Citrus sinensis* (EO)** | | Pure | | 100 | | 1.74 (1.65-1.82) | | 2.73 (2.58-2.90) | | 2.72 | | 5 | | 0.77 | |
|  |  | Emulsion | | 100 | | 2.48 (2.42-2.64) | | 4.27 (3.95-4.69) | | 6.50 | | 4 | | 0.19 | |
| ***D-Limonene*** | | Pure | | 100 | | 0.55 (0.50-0.60) | | 1.117 (0.99-1.26) | | 1.76 | | 3 | | 0.62 | |
|  |  | Emulsion | | 100 | | 2.50 (2.40-2.60) | | 3.35 (3.18-3.60) | | 4.6 | | 4 | | 0.20 | |
| ***Citrus sinensis* (EO)** | | High Energy | | 100 | | 1.94 (1.85-2.02) | | 2.78 (2.64-2.96) | | 1.4 | | 4 | | 0.84 | |
|  |  | Low Energy | | 100 | | 3.39 (3.28-3.50) | | 4.57 (4.35-4.86) | | 3.79 | | 4 | | 0.43 | |
| ***D-Limonene*** | | High Energy | | 100 | | 2.12 (2.04-2.19) | | 2.84 (2.73-2.98) | | 1.60 | | 4 | | 0.80 | |
|  |  | Low Energy | | 100 | | 2.84 (2.74-2.93) | | 3.67 (3.50-3.92) | | 3.20 | | 3 | | 0.36 | |
| LC₅₀ and LC₉₀: lethal concentrations that kill 50 % and 90% of tested individuals, respectively. The values between parentheses present the lower and upper concentration limits, corresponding to the 95 % Fiducial intervals (F.I); χ2: Chi-square test; df: degree of freedom; P: test probability. | | | | | | | | | | | | | | | |

| **Supplementary Table 2**. Detailed effect of different preparations of *Citrus sinensis* essential oil and D-limonene on the parasitism of *Pachycrepoideus vindemmiae* | | | | | |
| --- | --- | --- | --- | --- | --- |
| **TRT** | **Lethal concentration** | **Parasitized^a^** | **Unparasitized and unemerged^b^** | **Unparasitized and emerged^c^** | **Parasitism (%)^d^** |
| **Negative Control** | **_** | 0 | 6 | 144 | 0 |
| **Positive Control** | **_** | 147 | 0 | 3 | 98.33 |
| **Tween** | **_** | 148 | 0 | 2 | 98.66 |
| **Buffer** | **_** | 148 | 2 | _ | 98.66 |
| ***C. sinensis* EO (pure)** | **LC₅₀** | 99 | 48 | 3 | 66* |
|  | **LC₉₀** | 105 | 45 | _ | 70* |
| **Limonene (pure)** | **LC₅₀** | 103 | 47 | _ | 68.66* |
|  | **LC₉₀** | 120 | 29 | 1 | 80 |
| ***C. sinensis* Emulsion** | **LC₅₀** | 108 | 42 | _ | 72* |
|  | **LC₉₀** | 113 | 34 | 3 | 75.33* |
| **Limonene Emulsion** | **LC₅₀** | 109 | 41 | _ | 72.66* |
|  | **LC₉₀** | 118 | 30 | 2 | 78.66 |
| ***C. sinensis* Low Energy** | **LC₅₀** | 118 | 32 | _ | 78.33 |
|  | **LC₉₀** | 100 | 46 | 4 | 66.66* |
| **Limonene Low Energy** | **LC₅₀** | 116 | 34 | _ | 77.33* |
|  | **LC₉₀** | 117 | 33 | _ | 78* |
| ***C. sinensis* High Energy** | **LC₅₀** | 137 | 12 | 1 | 91.33 |
|  | **LC₉₀** | 105 | 44 | 1 | 70* |
| **Limonene High Energy** | **LC₅₀** | 112 | 38 | _ | 74.66* |
|  | **LC₉₀** | 101 | 45 | 4 | 67.33* |
| ( ^a^ ): Parasitized indicates pupae parasitized by *P. v*indemmiae and characterized by the presence of an emergence hole, or pupae parasitized by *P. vindemmia*e but did not result in emergence in these cases, when the larval parasitoid developed a noticeable gap formed between the parasitoid pupa and the puparium shell, making the entire parasitoid pupa visible under a microscope. ( ^b^ ): Unparasitized and unmerged indicates pupae were fully intact without signs of *D. suzukii* emergence. ( ^c^ ): Unparasitized and emerged indicates puparium showing signs of successful emergence and presence of *D. suzukii* flies. ^( d )^: Parasitism rate estimated as % parasitized pupae (N =150). (*) indicate a significant statistical difference using ANOVA on Ranks (Kruskal-Wallis test) and Dunn's (p < 0.05) as a post-hoc test. | | | | | |
